# Positive Association between Indoor Gaseous Air Pollution and Obesity: An Observational Study in 60 Households

**DOI:** 10.3390/ijerph182111447

**Published:** 2021-10-30

**Authors:** Jia-Kun Chen, Charlene Wu, Ta-Chen Su

**Affiliations:** 1 Institute of Environmental and Occupational Health Sciences, College of Public Health, National Taiwan University, Taipei 10055, Taiwan; jkchen29@ntu.edu.tw; 2 Global Health Program, College of Public Health, National Taiwan University, Taipei 10055, Taiwan; charlenewu@ntu.edu.tw; 3 Department of Environmental and Occupational Medicine, National Taiwan University Hospital, Taipei 100225, Taiwan; 4 Department of Internal Medicine and Cardiovascular Center, National Taiwan University Hospital, Taipei 100225, Taiwan

**Keywords:** indoor air pollution, carbon monoxide, total volatile organic compounds, gas emission, obesity

## Abstract

This study aims to analyze whether exposure to indoor air pollution affects obesity. In our research, we recruited 127 participants, with an average age of 43.30 ± 15.38 years old, residing in 60 households. We monitored indoor air quality for 24 h, and conducted both questionnaire surveys and collected serum samples for analysis, to assess the relationship between indoor air pollutant exposure and obesity. After adjusting for demographic characteristics, the results showed that CO_2_ exposure is positively associated with being overweight and with a higher risk of being abdominally obese. Exposures to CO and formaldehyde were also positively associated with being overweight. IQR increase in TVOC was positively associated with increases in the risk of a high BMI, being abdominally obese and having a high body fat percentage. Two-pollutant models demonstrate that TVOCs presented the strongest risks associated with overweightness. We concluded that persistent exposure to indoor gaseous pollutants increases the risk of overweightness and obesity, as indicated by the positive association with BMI, abdominal obesity, and percentage body fat. TVOCs display the strongest contribution to obesity.

## 1. Introduction

Estimates published by the World Health Organization (WHO) in 2016 revealed that worldwide prevalence of obesity has tripled since 1975, with nearly 13% of adults being obese; such prevalence is estimated to reach 18% for men and 21% for women by 2025 [1,2]. Recent literature substantiated obesity as one of the leading causes of noncommunicable diseases, including cardiovascular diseases, diabetes, musculoskeletal disorders, and cancers [3,4,5]. Even though the cardinal cause of obesity is accepted to be due to an energy imbalance between calories intake and expenditure, the underlying mechanism of such a condition actually involves multifactorial interactions between genetic and non-genetic risk factors [6,7]. In the past decade, increasing interest has focused on the roles that environmental risk factors have on the continued rise in obesity prevalence [8,9,10], with air pollutants as suspected environmental obesogens [8,11,12].

Exposure to ambient air pollutants poses adverse health effects on the general population. Specifically, particulate matter (PM) and volatile organic compounds (VOCs) have been associated with elevated blood pressure, decreased heart rate variability, and increased systemic inflammation and oxidative stress, all of which can lead to cardiovascular morbidity and mortality [13,14]. Previous investigations reported that air pollution can elicit oxidative stress and inflammation, both of which result in metabolic dysfunction and contribute to the development of insulin resistance and, thus, increase susceptibility to diabetes [15,16]. Behaviorally, poor air quality decreases the desire to exercise outdoors and thus results in restricted physical activity [17,18]. Additionally, numerous other studies provided evidence demonstrating exposure to outdoor air pollutants is associated with increased risk of obesity [19,20,21,22,23].

Since humans spend up to 87% of time indoors, and outdoor sources of air pollutant can penetrate buildings, indoor air quality can be influenced concurrently by ambient sources [24]. The WHO reported that the hazardous characteristics of indoor PMs are similar to those found outdoors; additionally, the indoor concentrations of PM are usually higher to those in the environment [25]. A study conducted by de Gennaro and associates demonstrated that most exposures to VOCs also occur indoors, some of these being classified as known or possible human carcinogens [26]. Existing literature has demonstrated exposure to indoor PM_2.5_ and total volatile organic compounds (TVOCs) can decrease heart rate variability and elevate blood pressure [27,28]; other work showed that improved indoor air quality using air conditioning or filters ameliorated systemic oxidative stress and inflammation, as indicated by biomarkers, such as high sensitivity-C-reactive protein (hs-CRP) and 8-hydroxy-2′-deoxyguanosine (8-OHdG) [13,29]. All such evidence demonstrates that adverse health effects resulting from indoor air pollution is of crucial importance.

However, limited research has focused on the adverse health effects of indoor air pollutants on body weight. Kermah and associates revealed that indoor secondhand smoke exposure increased the risk of obesity in children [30]. On the other hand, Rajkumar and associates’ research concluded that indoor PM_2.5_ and black carbon exposure were not found to correlate with abdominal obesity in women [31]. Therefore, our study aims to evaluate the association between exposure to household air pollutants and obesity among young and middle-aged adults.

## 2. Materials and Methods

### 2.1. Study Subjects

**Figure 1:**
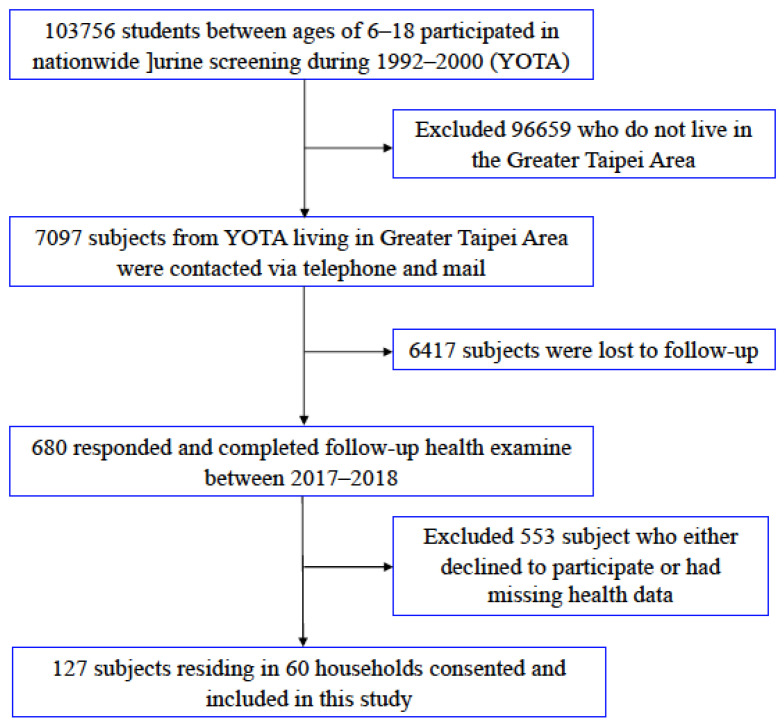
Flowchart of study participants in the Young Taiwanese (YOTA) cohort; with a total of 127 subjects enrolled.

Participants originated from the previously established Young Taiwanese Cohort (YOTA) from 2006–2008 to investigate the effects that childhood cardiovascular risk factors have on later life [32]. With approval obtained from the Institutional Review Board (IRB) of National Taiwan University Hospital (NTUH), we contacted the 886 participants from our previous study via telephone and mail according the telephone number or address submitted. Among them, 680 young adults from 2017–2018 consented to complete follow-up health examinations and responded to a questionnaire on individual demographic information, as well as lifestyle choices, such as smoking, drinking, and exercise. In the end, 127 participants residing in 60 households consented to take part in our study and allowed in-house air quality monitoring. A diagram denoting the process through which the study subjects were recruited is presented in Figure 1.

### 2.2. Cardiovascular Risk Factors Assessment and Serum Analysis

The health examination included a clinician interview, a self-reported questionnaire and venous blood biochemical analysis. The interview with a clinician recorded demographic information, such as age, sex, height, and body weight, as well as family medical history of CVD, hyperlipidemia, diabetes mellitus (DM), and hypertension. Participants responded to questions related to socioeconomic status and lifestyle choices, including but not limited to smoking, alcohol consumption, and physical exercise. Systolic and diastolic blood pressures (SBP and DBP) were measured using a mercury sphygmomanometer in a standardized fashion, once on each arm, with a 5-min of rest between measurements. If the difference in the two measurements was greater than 10 mm Hg, then a third measurement was obtained; the final recorded BP was the average of the lowest two measured BP values. Furthermore, we defined hypertension as a history of hypertension, prescribed with anti-hypertensive medication, or with SBP > 140 mmHg and/or DBP > 90 mmHg. Type 2 diabetes mellitus status was defined as a prior physician diagnosis or those using oral hypoglycemic agents or who had a measured fasting glucose ≥126 mg/dL.

The BMI was calculated using the formula: weight (kg)/[height (m)]^2^. Body composition examination was performed by bioelectrical impedance analysis (Tanita, Model MC-780MA, Tokyo, Japan). A serum blood sample was collected from an antecubital vein after an overnight fasting period of 10–14 h. The blood glucose, serum total cholesterol, triglyceride, and high- and low-density lipoprotein cholesterol (HDL-C and LDL-C) were analyzed using an auto-analyzer (Hitachi 7250 Special; Hitachi, Tokyo, Japan) at the central lab at NTUH.

### 2.3. 24-h Continuous Indoor Air Quality Monitoring

The indoor air quality of the 60 households in which participants reside was monitored continuously for 12 h (PM 06:00 to AM 06:00 CST the next day) and 24 h to clearly demonstrate the real-time range of exposure concentrations; the 12 h timeframe was chosen to represent the hours in which residents were are home. Monitors were set up in the living rooms, the real-time concentrations of total suspended particle (TSP) and PM (PM_1.0_, PM_2.5_, PM_4.0_, PM_10_), gaseous pollutants (CO_2_, CO, TVOCs, and formaldehyde), temperature, and relative humidity were documented during the summer season of 2017–2018. TSP and PM levels were measured with an aerosol monitor (DustTrak DRX Model 8533, TSI Incorporated, Shoreview, MN, USA). A multifunctional Q-Track monitor tracked the concentration of CO and CO_2_ (TSI^®^ Model 7575, TSI Incorporated, Shoreview, MN, USA); TVOCs were monitored using a portable handheld VOC monitor (ppbRAE 3000, Honeywell, San Jose, CA, USA). Formaldehyde was monitored continuously (Formaldemeter™ htV-M; PPM Technology Ltd., Caernarfon, Wales, UK).

### 2.4. Statistical Analyses

With the monitoring time duration separated into two intervals, the 12-h and 24-h averages of the exposure concentrations for six air pollutants (PM_2.5_, PM_10_, TVOC, formaldehyde, CO, and CO_2_) were expressed as quartiles, along with the interquartile range (IQR). For the basic characteristics, the continuous variables were expressed as mean ± SD, and the binary variables were presented as percentages.

Partial correlation analysis of the six air pollutions was applied to estimate the strength and direction of association between the pollutants under examination and body composition, after controlling for age, gender, fasting glucose level, health behavior characteristics (smoking habit, alcohol consumption, incense burning, exercise habit, sleep deprivation, diet supplement, and frequency of home cooked meals), and socioeconomic status (education level, marital status, employment type), all of which potentially contribute to obesity. Furthermore, adjusted multiple logistic regression models were used to assess the association between exposures to IQR increases in air pollutant concentrations and the following obesity-related factors: overweight, abdominal obesity, and fat percentage. Both 12-h and 24-h exposure durations were examined. In addition to our crude model with no adjustment (Model 1), the second model (Model 2) adjusted for age and gender. The third model (Model 3), controlled for age, gender fasting glucose level, as well as aforementioned health behavior and socioeconomically-related covariates. Finally, two pollutant models were used to analyze the intensity of the effects of the two pollutants on obesity-related factors, after controlling for previously mentioned covariates that could affect obesity. Effects of multicollinearity were examined by calculating variance inflation factor (VIF).

All analyses were performed using SAS software (version 9.4; SAS Institute, Cary, NC, USA). Statistical significance was determined based on a *p* value < 0.05 with a two-tail distribution.

## 3. Results

The basic demographic characteristics of our study participants are listed in Table 1. These residents were stratified according to BMI status. Of the 127 participants, 38 were considered overweight or obese (BMI ≥ 25) and 89 had normal body weight (BMI < 25). Our study subjects consisted of a working age population, and the respective mean ages of those who were overweight/obese and those with normal weights were 41.84 and 43.92 years. Furthermore, more than half of the overweight/obese participants were male (57.89%). Regardless of BMI status, the majority of our cohort were never-smokers, consisting of 26 (68.42%) overweight individuals and 76 (85.39%) who possessed normal weights. Similar proportions of participants identified as nondrinkers. Furthermore, health-related behaviors, such as smoking and incense burning statistically significantly affected BMI status. Significant correlations were evident for waistline, blood pressures (SBP and DBP), total cholesterol, and diabetic status between overweight and normal body weight individuals.

**Figure 2:**
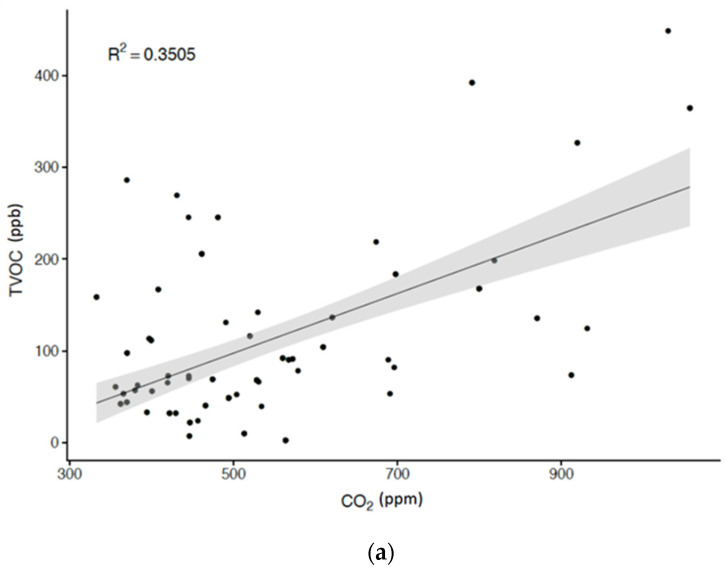

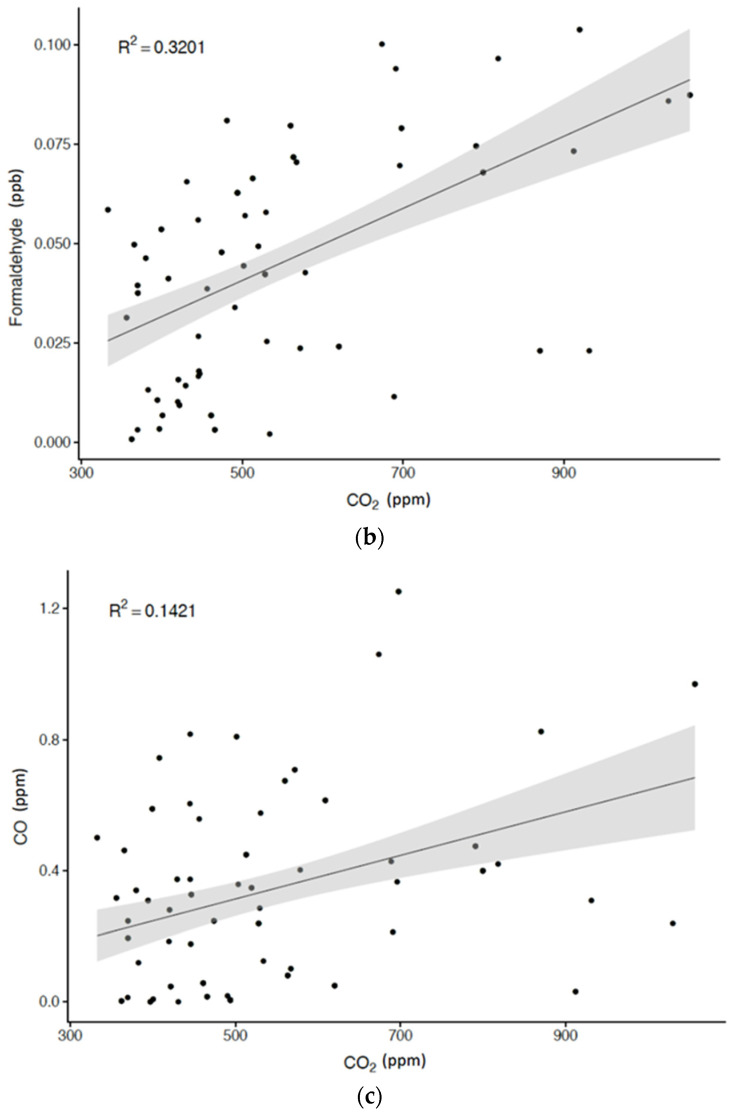
Scatter diagram of the concentration of (**a**) carbon monoxide (CO); (**b**) formaldehyde (CH_2_O); and (**c**) total volatile organic compounds (TVOCs) in relation to carbon dioxide (CO_2_).

We documented the 12- and 24-h average concentrations of the air toxins monitored in Table 2. The 12-h arithmetic mean concentrations of PM_2.5_, PM_10_ and TVOC were 27.61, 28.74 and 112.48 μg/m^3^, which were comparable to those of 24 h. The mean and quartile concentrations of TVOCs and CO_2_ after 12 h of monitoring were consistently greater than those of 24 h. After adjusting for covariates, such as age, gender, health behaviors, and socioeconomic status, the associations between the indoor levels of TVOCs, formaldehyde and CO to CO_2_ are illustrated in Figure 2. We found that concentrations of TVOCs and formaldehyde increased linearly with CO_2_, and that such an association was statistically significant, with coefficients of determination of 0.3505 and 0.3201 (Figure 2a,b). A less strong, but still statistically significant correlation, exists between CO and CO_2_ (r^2^ = 0.1421; Figure 2c). In Table 3, additional partial correlation analyses indicated that CO, CO_2_, formaldehyde, and TVOCs were positively associated with obesity measurements, including BMI, waistline, body fat percentage, fat mass, and visceral fat. Our results demonstrated that TVOCs displayed the strongest correlation to the aforementioned obesity indicators, followed by CO_2_, CO, and formaldehyde.

Table 4 shows the estimated odds ratios (OR) of becoming obese, as indicated by BMI, abdominal obesity, and high body fat percentage (defined as ≥32%), after 12-h exposure to air pollutants, monitored according to different model specifications. Results demonstrated that exposure per IQR increases in concentrations of PM_2.5_ and PM_10_ is not associated with statistically significant increases in risk of overweightness (BMI ≥ 25), abdominal obesity, or fat percentage, regardless of model adjustments. In our crude model without adjustment (Model 1), every IQR increase in exposure concentrations to CO was associated with an increased risk of becoming overweight, as indicated by an OR of 1.71 (95% confidence interval (CI): 1.00, 2.91). A similar statistically significant association was evident with body fat percentage (OR = 1.92, 95% CI: 1.11, 3.35). IQR exposure to CO_2_ is associated with higher risk of overweightness (OR = 1.77, 95% CI: 1.17, 2.66), abdominal obesity (OR = 1.61, 95% CI: 1.08, 2.40), and high body fat percentage (OR = 1.51, 95% CI: 1.00, 2.27). Our second model that adjusted for age and gender (Model 2) demonstrated that per IQR increase in exposure to TVOC was associated with higher risks of overweightness (OR = 2.14, 95% CI: 1.33, 3.44) and abdominal obesity (OR = 1.97, 95% CI: 1.25, 3.11). Similar positive associations were evident with exposure to formaldehyde and CO_2_.

Furthermore, we also note that per IQR increase in exposure to CO is associated with higher risks of high BMI (OR = 1.86, 95% CI: 1.06, 3.25) and high body fat percentage (OR = 2.17, 95% CI: 1.51, 4.10). Our final model adjusted for age, gender, fasting glucose level, health behavior characteristics, and socioeconomic status (Model 3); we found that risks of overweightness became amplified for TVOCs, formaldehyde, CO, and CO_2_, with ORs of 2.70 (95% CI: 1.48, 4.94), 2.69 (95% CI: 1.05, 4.94), 2.25 (95% CI: 1.14, 4.44), 2.46 (95% CI: 1.41, 4.29), respectively. Most notably, exposure to TVOCs was associated with higher odds of abdominal obesity (OR = 2.38, 95% CI: 1.28, 4.40) and percentage body fat (OR = 2.09, 95% CI: 1.09, 3.99). Likewise, exposure to CO_2_ was also shown to be associated with statistically significantly higher risks of overweightness, abdominal obesity, and high body fat percentage.

Results of our two-pollutant models are presented in Table 5; it is evident that formaldehyde ceases to be the stronger predictor for all three indicators of obesity, regardless of the two-pollutant combinations. When CO_2_ is placed in a model combination with either formaldehyde or CO, CO_2_ remained as the stronger contributor to overweightness, with an adjusted OR (aOR) of 2.37 (95% CI: 1.24, 4.54) and1.86 (95% CI: 1.01, 3.44), respectively. However, TVOC outweighs formaldehyde and CO as the stronger predictor of overweightness, as indicated by aORs of 2.60 (95% CI: 1.32, 5.31) and 2.22 (95% CI: 1.13, 4.35). For abdominal obesity, TVOC was the stronger predictor in models with two-pollutant combinations of CO_2_, formaldehyde and CO. For high body fat percentage, our two-pollutant model with CO_2_ and formaldehyde shows that CO_2_ was a stronger predictor of high body fat percentage, with aOR of 2.21 (95% CI: 1.09, 4.47); however, the predictive power of CO_2_ diminished when compared to CO. Our analysis demonstrated indoor exposure to per IQR of CO, when compared to CO_2_, TVOC, and formaldehyde, is correlated with high body fat percentage, as indicated by adjusted ORs of 2.75, 3.69, and 4.46, respectively.

## 4. Discussion

The results from our pilot study indicate that indoor gaseous air pollution is positively associated with overweightness, abdominal obesity, and high body fat percentage. Our investigation continuously monitored the household levels of PM_2.5_, PM_10_, CO, CO_2_, formaldehyde and TVOCs for 12 and 24 h. These concentrations demonstrated the degree to which residents’ time–activity patterns can affect participant exposure. Since residential time–activity patterns are unlikely to undergo drastic changes, the monitoring of indoor air quality can serve to indicate long-term exposure in a household; previous studies have applied similar logic [33,34]. Specifically, our results identified CO, CO_2_, formaldehyde and TVOCs are associated with higher risks of attaining overweightness, developing abdominal obesity or obtaining high percentage body fat with varying strength. Per IQR increase in exposure concentration to indoor TVOC, in particular, is most significantly correlated with higher risks of overweightness and abdominal obesity.

Previous studies exploring the association between exposure to air pollution and obesity released conflicting results. Certain literature found air pollution to correlate with BMI levels and obesity in children; another longitudinal study observed synergistic effects of near-road air pollution and second-hand smoke on BMI status [19,20]. However, the aforementioned studies omitted which types of air toxics contributed to the observed positive association with obesity. Our findings suggest there is no association between indoor exposure to PM_2.5_ and PM_10_ and overweightness, which is consistent with the conclusions reached by White and colleagues, where exposure to ambient PM_2.5_ did not demonstrate significant association to weight gain in adult African-American females [35]. Furthermore, Walkwork and team showed chronic exposure to low ambient PM_2.5_ levels (ranged between 4.2 and 13.6 μg/m^3^) did not result in an increased risk for developing abdominal obesity [36]. Our results also complement the results reported on Spanish school children, where exposures to indoor PM_2.5_ at both home and school fall short of establishing a positive association with BMI [37]. It is worth noting that studies conducted in China and Southern Korea documented positive associations between ambient PM and risk of obesity in children and adults [22,23]; however, the exposures of outdoor PM were either much higher compared to that of our participants.

Although Danish scholars first proposed that at atmospheric CO_2_ promotes obesity, the findings fall short of confirming such a connection [38]. Another study demonstrated that increasing trends in obesity and diabetes corresponds to increasing ambient CO_2_ levels, but such correlation did not attain statistical significance [39]. Our multiple logistic regression analysis demonstrated that residential CO_2_ levels are positively associated with higher risk of overweightness and abdominal circumference, both of which can lead to obesity and manifest clinically as metabolic syndrome. Such results identified CO_2_ as an important obesity-related indoor air pollutant, which also is a well-known index of indoor air ventilation. In building physics, indoor CO_2_ concentration is indicative of air quality, ventilation efficiency, air exchange rates and has been associated with a higher risk of sick building syndromes and allergies [40,41].

Household concentrations of CO typically originate from cooking activities, due to incomplete combustion of biofuels such as natural gas and kerosene. The toxic effects from CO exposure can manifest in the heart and cardiovascular systems, resulting in myocardial ischemia and increased cardiac arrhythmias [42,43,44]. However, insufficient research focus on the association between CO exposure and overweightness. Although the 12- and 24-h mean concentrations of indoor CO were 0.34 and 0.37 ppm, both of which fall below the guidelines recommended by WHO [45], our analyses show CO is a stronger predictor for high body fat percentage across all model combinations. Since the majority of our participants identified as non-smokers, the indoor concentration of CO, albeit low, we attribute the presence of CO to cooking and to the burning of incense (Table 1). Additionally, natural gas boilers could be another contributing source of high CO levels, since they are installed in the majority of households in our investigation. Therefore, lifestyle choices and health-related habits can contribute to increases in indoor air pollutant concentrations, thus leading to higher chances of obesity.

Most interestingly, we observed significant associations between indoor concentrations of TVOCs and risk of increasing body weight, abdominal circumference and percentage of body fat. We assessed these associations with TVOCs in two-pollutant models; after adjusting for covariates including age, gender, and health-related behavior habits, such positive correlations remain for the other two-pollutant combinations. Furthermore, our model indicated that presence of multicollinearity is low across all models, as indicated by VIF. These findings indicated that exposure to indoor TVOCs magnifies the risk of obesity, independent of poor indoor ventilation and other gaseous pollutants. Since household emissions of VOCs originate from passive emissions from paints and adhesives in furniture, the use of care products, such as laundry detergent or from cooking activities, further research examining which specific VOCs in households are correlated to increased risk of overweightness and obesity.

## 5. Study Limitations

The experimental limits of this study should be disclosed. Firstly, the current results have yet to reflect how household air pollution levels may vary from season to season, as our sampling time took place in the summer (July–September of 2017 and 2018). Secondly, results from our observational cohort study elucidated on association and not causality. Furthermore, specific constituents of PM, such as bioaerosols and heavy metals, should be evaluated in future studies. Moreover, the results of our findings may not be generalized to specific population subgroups, such as toddlers, the elderly, or those suffering from chronic diseases. Finally, the 127 participants lived in different indoor environments and work settings, as well as had different diet intakes for the duration of this investigation. Since these aforementioned variables were not controlled, the degree to which these variables affect the study outcome is difficult to evaluate. Future study could also consider recruiting more participants to ensure statistical power.

## 6. Conclusions

After adjusting for demographic characteristics, an interquartile range (IQR) increase in exposure to CO_2_ (554.95 ppm) is positively associated with being overweight, indicated by a higher risk of high body mass index (BMI ≥ 25 kg/m^3^) with an adjusted odds ratio (aOR) of 2.46 (95% CI: 1.41–4.29) and also a higher risk of becoming abdominally obese (aOR = 1.67; 95% CI: 1.01–2.76); exposures to CO and formaldehyde were also positively associated with being overweight. IQR increase in TVOCs is positively associated with increases in risk of high BMI (aOR = 2.70; 95% CI: 1.48, 4.94), becoming abdominally obese (aOR = 2.38; 95% CI: 1.28, 4.40), and obtaining high body fat percentage (aOR = 2.09; 95% CI: 1.09, 3.99). Two-pollutant models, which were used to determine which air pollutant has more influence on the adverse health outcome, demonstrate that TVOCs present the strongest risks associated with overweightness.

The present study established that indoor concentrations of CO, CO_2_, formaldehyde, and TVOCs by continuous monitoring is associated with indicators of obesity despite the abovementioned limitations. The adverse health effects associated with poor indoor air quality (IAQ) have been well documented [46,47,48,49]. Since people spend the majority of their lifetimes indoors, continued research on identifying specific components of TVOCs and whether biological pollutants contribute to obesity is warranted.

## Figures and Tables

**Table 1:** Basic characteristics of 127 subjects living in 60 households.

	BMI ≥ 25 (*N* = 38)	BMI < 25 (*N* = 89)	*p*-Value
	Mean ± SD or *n* (%)		
Age (years)	41.84 ± 14.13	43.92 ± 15.89	0.4869
Male gender; no. (%)	22 (57.89)	29 (32.58)	0.0077
Marital status; no. (%) Married Single	20 (52.63) 18 (47.37)	57 (64.04) 32 (35.96)	0.2280
Employment type, %			0.0873
Blue-collar worker	20 (52.63)	45 (50.56)	
White-collar worker	7 (18.42)	6 (6.74)	
BMI (kg/m^2^)	28.61 ± 2.89	21.65 ± 2.27	<0.0001
Waistline (cm)	95.58 ± 8.40	77.64 ± 8.18	<0.0001
Systolic BP (mmHg)	124.38 ± 13.20	118.34 ± 14.77	0.0314
Diastolic BP(mmHg)	72.19 ± 9.33	66.50 ± 9.10	0.0017
Hypertension; no. (%)	8 (21.05)	11 (12.36)	0.2085
Cholesterol (mg/dL)	173.47 ± 33.31	199.93 ± 44.9	0.0004
LDL (mg/dL)	109.11 ± 31.92	129.55 ± 72.69	0.0296
HDL (mg/dL)	46.42 ± 9.99	61.95 ± 18.89	<0.0001
Triglyceride (mg/dL)	134 ± 83.28	105.63 ± 102.22	0.1337
Glucose AC (mg/dL)	96.11 ± 32.22	93.08 ± 34.65	0.6462
Diabetes; no. (%)	11 (28.95)	12 (13.48)	0.0382
Smoking habit, no. (%)			0.0500
Never	26 (68.42)	76 (85.39)	
Former	4 (10.53)	7 (7.87)	
Current	8 (21.05)	6 (6.74)	
Alcohol consumption, no. (%)			0.6306
Never	26 (68.42)	63 (70.79)	
Former	5 (13.16)	7 (7.87)	
Current	7 (18.42)	19 (21.35)	
Exercise habit, %	12 (31.58)	38 (42.70)	0.2403
Incense burning, %			0.0353
Everyday	19 (50.00)	24 (27.27)	
Not Everyday	2 (5.26)	12 (13.64)	
Never	17 (44.74)	52 (59.09)	
Education level, %			0.9396
College or graduate school	10 (26.32)	24 (26.97)	
High school or below	28 (73.68)	65 (73.03)	
Sleep deprivation; no. (%)	15 (39.47)	33 (37.08)	0.0873
Diet supplement; no. (%)	17 (44.74)	48 (53.93)	0.3424
Cook at home; no. (%)	36 (94.74)	85 (95.51)	-

**Table 2:** The 12 h and 24 h measurements indoor air quality in 60 households.

		Mean ± SD	Median	IQR	Range (Min to Max)
PM_2.5_, μg/m^3^	12 h	27.61 ± 16.42	23.40	22.41	86.06 (8.44–94.50)
	24 h	31.30 ± 16.50	27.72	24.03	92.38 (8.72–101.10)
PM_10_, μg/m^3^	12 h	28.74 ± 16.81	23.80	22.83	94.93 (0.04–94.97)
	24 h	33.06 ± 16.74	29.76	23.27	92.41 (10.44–102.85)
CO, ppm	12 h	0.34 ± 0.30	0.31	0.42	1.25 (0.00–1.25)
	24 h	0.37 ± 0.30	0.31	0.46	1.12 (0.00–1.12)
CO_2_, ppm	12 h	544.95 ± 174.63	493.98	188.97	723.92 (332.64–1056.56)
	24 h	533.54 ± 155.48	487.82	169.71	750.76 (359.12–1109.88)
TVOC, ppb	12 h	112.48 ± 96.50	78.13	109.91	445.51 (2.86–448.37)
	24 h	99.79 ± 84.22	71.11	76.06	425.92 (2.09–428.01)
Formaldehyde, ppb	12 h	4.47 ± 2.82	4.53	5.06	10.28 (0.09–10.37)
	24 h	4.57 ± 2.62	4.95	5.02	9.63 (0.37–10.00)

**Table 3:** Partial correlation coefficient between gaseous air pollutants and obesity measurements obtained from 127 participants in 12 h.

Obesity Indicators (Unit)	TVOC (ppb)	Formaldehyde (ppm)	CO (ppm)	CO_2_ (ppm)
Body Mass Index (kg/m^2^)	0.3925 ^‡^	0.1947 *	0.2249 *	0.2941 ^†^
Waistline (cm)	0.3434 ^‡^	0.1897 *	0.1272	0.2290 *
Fat Percentage (%)	0.3267 ^‡^	0.2571 ^†^	0.2087 *	0.2833 ^†^
Fat Mass (kg)	0.3354 ^‡^	0.2084 *	0.1798	0.2614 ^†^
Fat Free Mass (kg)	0.2852 ^†^	0.1498	0.1109	0.1811
Muscle Mass (kg)	0.2867 ^†^	0.1492	0.1080	0.1811
Visceral Fat Rating	0.3353 ^‡^	0.2239 *	0.2036 *	0.2498 ^†^

Adjusted for age, gender, fasting glucose level, health behaviors (smoking habit, alcohol consumption, incense burning, exercise habit, sleep deprivation, diet supplement and frequency of homecooked meals), and socioeconomic status (education level, marital status, employment type). * Statistical significance set at *p*-value < 0.05 *, *p*-value < 0.01 ^†^, *p*-value < 0.001 ^‡^.

**Table 4:** Estimated odds ratio per IQR increase for the risk of obesity after 12 h of exposure to indoor gaseous pollutants.

	Overweight (BMI ≥ 25 kg/m^3^)		Abdominal Obesity (Male ≥ 90 cm, Female ≥ 80 cm)		Fat Percentage ≥ 32%	
Model 1	OR (95%CI)	*p*-value	OR (95%CI)	*p*-value	OR (95%CI)	*p*-value
PM_2.5_	0.87 (0.50, 1.53)	0.6396	0.80 (0.47, 1.35)	0.3993	1.13 (0.66, 1.94)	0.6538
PM_10_	0.91 (0.52, 1.57)	0.7285	0.75 (0.44, 1.28)	0.2976	1.16 (0.68, 1.98)	0.5913
CO	1.71 (1.00, 2.91)	0.0482	1.14 (0.68, 1.88)	0.6231	1.92 (1.11, 3.35)	0.0206
CO_2_	1.77 (1.17, 2.66)	0.0065	1.61 (1.08, 2.40)	0.0197	1.51 (1.00, 2.27)	0.0491
TVOC	2.05 (1.30, 3.22)	0.0020	1.95 (1.24, 3.06)	0.0039	1.45 (0.94, 2.23)	0.0929
Formaldehyde	2.25 (1.09, 4.66)	0.0291	1.74 (0.89, 3.42)	0.1055	1.51 (0.73, 3.16)	0.2693
Model 2						
PM_2.5_	0.90 (0.52, 1.56)	0.6976	0.80 (0.46, 1.37)	0.4104	1.07 (0.57, 1.98)	0.8374
PM_10_	0.92 (0.53, 1.59)	0.7596	0.75 (0.43, 1.30)	0.3022	1.13 (0.62, 2.07)	0.6861
CO	1.86 (1.06, 3.25)	0.0297	1.12 (0.67, 1.86)	0.6699	2.17 (1.15, 4.10)	0.0170
CO_2_	1.90 (1.23, 1.23)	0.0036	1.62 (1.08, 2.43)	0.0186	1.55 (0.98, 2.43)	0.0586
TVOC	2.14 (1.33, 3.44)	0.0016	1.97 (1.25, 3.11)	0.0036	1.59 (0.97, 2.61)	0.0665
Formaldehyde	2.20 (1.03, 4.69)	0.0405	1.82 (0.92, 3.59)	0.0863	1.85 (0.83, 4.12)	0.1342
Model 3						
PM_2.5_	0.87 (0.44, 1.71)	0.6806	0.97 (0.50, 1.92)	0.9402	1.18 (0.53, 2.64)	0.6858
PM_10_	0.90 (0.46, 1.74)	0.7463	0.92 (0.47, 1.78)	0.7956	1.28 (0.59, 2.81)	0.5344
CO	2.25 (1.14, 4.44)	0.0191	1.20 (0.65, 2.21)	0.5639	3.39 (1.34, 8.57)	0.0098
CO_2_	2.46 (1.41, 4.29)	0.0015	1.67 (1.01, 2.76)	0.0460	1.88 (1.06, 3.33)	0.0310
TVOC	2.70 (1.48, 4.94)	0.0012	2.38 (1.28, 4.40)	0.0059	2.09 (1.09, 3.99)	0.0231
Formaldehyde	2.69 (1.05, 6.85)	0.0386	1.48 (0.64, 3.40)	0.3565	1.48 (0.56, 3.94)	0.4335

Model 1: crude model with no adjustment. Model 2: adjusted for age and gender. Model 3: adjusted for age, gender, fasting glucose level, health behavior characteristics (smoking habit, alcohol consumption, incense burning, exercise habit, sleep deprivation, diet supplement and frequency of homecooked meals), and socioeconomic status (education level, marital status, employment type).

**Table 5:** Adjusted odds ratio of obesity indices and exposure to indoor gaseous pollutants in two-pollutant model (12-h exposure).

		Overweight (BMI ≥ 25 kg/m^3^)			Abdominal Obesity (Male ≥ 90 cm, Female ≥ 80 cm)			Fat Percentage ≥ 32% (≥75th Percentile)		
Pollutants (IQR)		aOR (95%CI)	*p*-Value	VIF	aOR (95%CI)	*p*-Value	VIF	aOR (95%CI)	*p*-Value	VIF
(1)	CO_2_	1.69 (0.84, 3.37)	0.1385	1.80	1.08 (0.58, 2.02)	0.8079	1.79	1.38 (0.68, 2.79)	0.3841	1.80
	TVOC	1.95 (0.94, 4.07)	0.0731	1.73	2.28 (1.12, 4.63)	0.0232	1.72	1.69 (0.76, 3.76)	0.1989	1.73
(2)	CO_2_	2.37 (1.24, 4.54)	0.0090	1.70	1.73 (0.95, 3.16)	0.0729	1.70	2.21 (1.09, 4.47)	0.0301	1.70
	Formaldehyde	1.17 (0.38, 3.63)	0.7858	1.67	0.86 (0.31, 2.42)	0.7796	1.66	0.67 (0.19, 2.31)	0.5237	1.67
(3)	CO_2_	1.86 (1.01, 3.44)	0.0480	1.50	1.59 (0.89, 2.86)	0.1177	1.50	1.63 (0.81, 3.30)	0.1805	1.50
	CO	1.68 (0.81, 3.50)	0.1621	1.29	0.94 (0.47, 1.87)	0.8623	1.29	2.75 (1.04, 7.29)	0.0418	1.29
(4)	TVOC	2.60 (1.32, 5.13)	0.0057	1.48	2.59 (1.31, 5.11)	0.0062	1.48	2.60 (1.12, 6.03)	0.0235	1.48
	Formaldehyde	1.25 (0.42, 3.70)	0.6841	1.50	0.76 (0.29, 2.03)	0.5878	1.50	0.59 (0.17, 2.09)	0.4164	1.50
(5)	TVOC	2.22 (1.13, 4.35)	0.0206	1.43	2.32 (1.13, 4.79)	0.0222	1.43	1.68 (0.77, 3.64)	0.1928	1.43
	CO	1.45 (0.69, 3.03)	0.3308	1.31	0.87 (0.42, 1.80)	0.7125	1.31	2.65 (1.00, 7.00)	0.0494	1.31
(6)	Formaldehyde	1.47 (0.47, 4.58)	0.5104	1.57	1.07 (0.38, 2.98)	0.9012	1.58	0.62 (0.17, 2.33)	0.4845	1.57
	CO	2.01 (0.93, 4.34)	0.0754	1.38	1.12 (0.56, 2.23)	0.7553	1.39	4.46 (1.50, 13.21)	0.0071	1.38

Multivariate logistic regression analyses after adjusted for age, gender fasting glucose level, health behavior characteristics (smoking habit, alcohol consumption, incense burning, exercise habit, sleep deprivation, diet supplement and frequency of homecooked meals), and socioeconomic status (education level, marital status, employment type).

## Data Availability

The authors choose to exclude this statement because the study did not report any data.
